# Ahr1 and Tup1 Contribute to the Transcriptional Control of Virulence-Associated Genes in Candida albicans

**DOI:** 10.1128/mBio.00206-20

**Published:** 2020-04-28

**Authors:** Sophia Ruben, Enrico Garbe, Selene Mogavero, Daniela Albrecht-Eckardt, Daniela Hellwig, Antje Häder, Thomas Krüger, Katrin Gerth, Ilse D. Jacobsen, Osama Elshafee, Sascha Brunke, Kerstin Hünniger, Olaf Kniemeyer, Axel A. Brakhage, Joachim Morschhäuser, Bernhard Hube, Slavena Vylkova, Oliver Kurzai, Ronny Martin

**Affiliations:** a Research Group Fungal Septomics, Leibniz Institute for Natural Product Research and Infection Biology—Hans Knoell Institute, Jena, Germany; b Research Group Host Fungal Interfaces, Septomics Research Centre, Friedrich Schiller University, Jena, Germany; c Leibniz Institute for Natural Product Research and Infection Biology—Hans Knoell Institute, Jena, Germany; d Department Microbial Pathogenicity Mechanisms, Leibniz Institute for Natural Product Research and Infection Biology—Hans Knoell Institute, Jena, Germany; e Biocontrol Jena GmbH, Jena, Germany; f Department Molecular and Applied Microbiology, Leibniz Institute for Natural Product Research and Infection Biology—Hans Knoell Institute, Jena, Germany; g Research Group Microbial Immunology, Leibniz Institute for Natural Product Research and Infection Biology—Hans Knoell Institute, Jena, Germany; h Institute of Microbiology, Friedrich Schiller University, Jena, Germany; i Institute for Molecular Infection Biology, University of Würzburg, Würzburg, Germany; j National Reference Center for Invasive Fungal Infections, Leibniz Institute for Natural Product Research and Infection Biology—Hans Knoell Institute, Jena, Germany; k Institute for Hygiene and Microbiology, University of Würzburg, Würzburg, Germany; University of Texas Health Science Center at San Antonio; Tel Aviv University

**Keywords:** *Candida albicans*, Tup1, filamentation, fungal virulence, gene regulation

## Abstract

The capacity of Candida albicans to reversibly change its morphology between yeast and filamentous stages is crucial for its virulence. Formation of hyphae correlates with the upregulation of genes *ALS3* and *ECE1*, which are involved in pathogenicity processes such as invasion, iron acquisition, and host cell damage. The global repressor Tup1 and its cofactor Nrg1 are considered to be the main antagonists of hyphal development in C. albicans. However, our experiments revealed that Tup1, but not Nrg1, was required for full expression of *ALS3* and *ECE1*. In contrast to *NRG1*, overexpression of *TUP1* was found to inhibit neither filamentous growth nor transcription of *ALS3* and *ECE1*. In addition, we identified the transcription factor Ahr1 as being required for full expression of both genes. A hyperactive version of Ahr1 bound directly to the promoters of *ALS3* and *ECE1* and induced their transcription even in the absence of environmental stimuli. This regulation worked even in the absence of the crucial hyphal growth regulators Cph1 and Efg1 but was dependent on the presence of Tup1. Overall, our results show that Ahr1 and Tup1 are key contributors in the complex regulation of virulence-associated genes in the different C. albicans morphologies.

## Abstract (precis)

Candida albicans is a major human fungal pathogen and the leading cause of systemic *Candida* infections. In recent years, Als3 and Ece1 were identified as important factors for fungal virulence. Transcription of both corresponding genes is closely associated with hyphal growth. Here, we describe how Tup1, normally a global repressor of gene expression as well as of filamentation, and the transcription factor Ahr1 contribute to full expression of *ALS3* and *ECE1* in C. albicans hyphae. Both regulators are required for high mRNA amounts of the two genes to ensure functional relevant protein synthesis and localization. These observations identified a new aspect of regulation in the complex transcriptional control of virulence-associated genes in C. albicans.

## INTRODUCTION

The major human fungal pathogen Candida albicans can grow in different morphologies: unicellular yeast cells, pseudohyphae, and (true) hyphae (1). This morphological plasticity is crucial for fungal pathogenicity as formation of hyphae is involved in adhesion to and invasion of host cells and tissues, while yeast cells are required for dissemination within the bloodstream (2, 3). Within host cells, hyphae and their associated proteins are involved in the acquisition of trace elements such as iron and zinc (4, 5). The different growth forms of C. albicans exhibit distinct interaction patterns with host immune cells. Yeast cells are recognized and taken up by macrophages. However, some cells have the potential to survive and start to form hyphae. These hyphae are able to destroy macrophages by early induction of pyroptosis, host glucose consumption, and toxin production and later direct escape by physical forces (6–9). In contrast, neutrophils are activated only by hyphae and are crucial for fungal killing (10). Consequently, neutropenic patients have a significantly impaired outcome for infections with C. albicans (11). Fungal polymorphism is also important for the interaction with dendritic cells but not natural killer cells (12, 13). In recent years, genes *ALS3* and *ECE1* were found to encode key contributors to fungal virulence (14, 15). Together with *DCK1*, *HGT2*, *HWP1*, *IHD1*, *RBT1*, and orf19.2457, they are also part of the core filamentation response (CFR), since their expression is induced whenever C. albicans forms hyphae, regardless of the environmental stimulus (16). *ALS3* encodes a multifunctional protein involved in adhesion, invasion, and iron acquisition (4, 14, 17). The *ECE1* product is normally the transcript with the highest abundance in C. albicans hyphae, independently of the environmental stimulus that triggered filamentation (16). The encoded protein is processed intracellularly by the proteinases Kex1 and Kex2 into eight peptides (15, 18, 19). One of these peptides, candidalysin, is secreted from hyphae into the environment and mediates host cell cytolysis (15). Candidalysin is involved in the immunopathology of *Candida* vaginitis (20) and drives protective innate type 17 cell responses during oral candidiasis (15, 21).

Due to their importance for fungal virulence, knowledge about the regulation of *ALS3* and *ECE1* expression can contribute to a better understanding of fungal pathogenicity mechanisms. Both genes are downregulated in mutants lacking key activators of filamentation (16, 22). In contrast, they are both upregulated in mutants lacking the hyphal growth repressors Tup1 and Nrg1 (23, 24). Despite this, *tup1*Δ and *nrg1*Δ mutants exhibit attenuated virulence in several infection models (23, 25–27).

Here, we show that the transcriptional regulators Ahr1 and Tup1 play crucial roles in the activation of *ALS3* and *ECE1*. While Tup1 contributes to repression and activation of the two genes, the presence of Ahr1 is important for high-level, functionally relevant expression of *ALS3* and *ECE1* in C. albicans hyphae.

## RESULTS

### Tup1 is required for activation of *ECE1* and *ALS3*.

**FIG 1:**
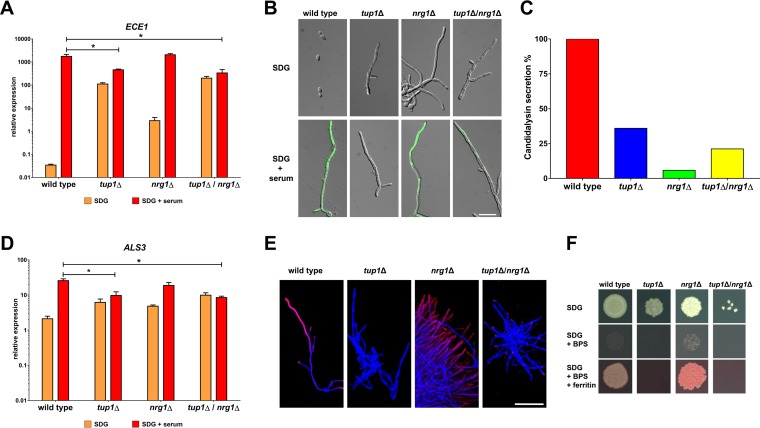
Tup1 is required for high-level expression of *ECE1* and *ALS3*. (A) Total RNA was isolated from the indicated strains after 6 h of growth in SDG with or without 10% human serum. *ECE1* transcription was normalized against *ACT1* and the control RNA (wild type, 5 h YPD, 37°C). Asterisks indicate significant transcription differences in a mutant compared to the wild type after growth in SDG with serum (*P* ≤ 0.05, two-tailed, unpaired Student's *t* test). (B) The indicated strains with integrated *pECE1-GFP* cassettes were grown for 6 h at 37°C in SDG with or without serum prior to microscopy. Shown are the overlays of the DIC channel and the GFP channel. Scale bar, 20 μm. (C) Candidalysin secretion was measured by LC-MS/MS after 18 h of growth in YNBS (pH 7.2). Candidalysin contents measured for wild-type hyphae were defined as 100%. (D) The total RNA isolated as described for panel A was used to determine the normalized relative expression levels of *ALS3*. Asterisks indicate significant transcription differences in a mutant compared to the wild type after growth in SDG with serum (*P* ≤ 0.05, two-tailed, unpaired Student's *t* test). (E) After 6 h of growth in SDG with 10% human serum at 37°C, cells of the indicated strains were stained first with a monoclonal anti-Als3 antibody (pink signal) and then with calcofluor white (blue signal). Overlays of the images taken in the Cy5 and DAPI (4′,6-diamidino-2-phenylindole) channel are shown. Scale bar, 50 μm. (F) From an overnight culture, 10^3^ cells of the indicated strains were dropped on SDG medium with or without an iron chelator (bathophenanthroline disulfonate [BPS]) and with or without ferritin. The plates were grown for 3 days at 37°C in 5% CO_2_ before images were taken.

We used RT-qPCR (reverse transcription followed by qPCR) to quantify the transcription of *ECE1* in the wild-type strain and in mutants lacking the hyphal growth repressors Nrg1 and Tup1 in minimal medium with or without 10% human serum. As expected, the gene was barely expressed in wild-type yeast cells but was highly upregulated in hyphae (Fig. 1A). It was expressed at higher levels in the filamentous mutants than in the wild type under yeast growth conditions, as expected (Fig. 1A). Under hyphal growth conditions, however, *ECE1* was expressed at significantly lower levels in the *tup1*Δ mutant than in the wild type, which was not the case for the *nrg1*Δ mutant (Fig. 1A). In fact, *ECE1* transcription in the *tup1*Δ mutant remained at an intermediate level under yeast and hyphal growth conditions. This unexpected low level of expression was not caused by Nrg1 activity as a *tup1*Δ/*nrg1*Δ double mutant showed levels of *ECE1* transcription similar to those seen with the *tup1*Δ single mutant (Fig. 1A). We used an established green fluorescent protein (GFP) reporter system for *ECE1* to visualize expression in these strains and observed that the level of upregulation of *ECE1* in the mutants under yeast growth conditions was not sufficient for a visible GFP signal (Fig. 1B). In accordance with the RT-qPCR data, *nrg1*Δ filaments showed bright GFP signals under hyphal growth conditions, while only weak fluorescence or no fluorescence was observed in the *tup1*Δ and *tup1*Δ/*nrg1*Δ mutants (Fig. 1B). The decreased *ECE1* expression in the *tup1*Δ mutant was not observed exclusively for serum-induced filamentation, as similar results were observed for hyphae which were induced by either pH or medium change (see Fig. S1 in the supplemental material). These results obtained with the GFP reporter system and RT-qPCR correlated with a significantly lower level of release of candidalysin by the *tup1*Δ mutant than by wild-type hyphae (Fig. 1C). However, we also noted that the *nrg1*Δ mutant was not able to release as much candidalysin as the wild type, indicating that some other defects in these mutants prevented the translation of high *ECE1* mRNA amounts into released candidalysin (Fig. 1C). *ECE1* is not the only core filamentation response gene which requires the presence of Tup1 for its high-level transcription in C. albicans hyphae. Similar expression patterns were observed for *ALS3* (Fig. 1D), *HWP1*, and *IHD1* but not for the other CFR genes (Fig. S2). As seen with *ECE1* and candidalysin, the intermediate levels of expression of *ALS3* in the *tup1*Δ and *tup1*Δ/*nrg1*Δ mutants were not sufficient for surface localization of the Als3 protein in these mutants (Fig. 1E). Both strains were also not able to use ferritin as an iron source; such use is known to be dependent on the presence of the Als3 protein on the cell surface (Fig. 1F) (3).

10.1128/mBio.00206-20.1 FIG S1 Tup1 is required for *ECE1* expression after pH shift and medium change. The wild-type strain and *tup1*Δ strains with integrated *pECE1-GFP* were grown in the indicated media for 6 h at 37°C prior to fluorescence microscopy. Shown are the overlays of the DIC channel and the GFP channels. Scale bar, 20 μm. Download FIG S1, TIF file, 1.7 MB. Copyright © 2020 Ruben et al. 2020 Ruben et al. This content is distributed under the terms of the Creative Commons Attribution 4.0 International license.

10.1128/mBio.00206-20.2 FIG S2 Core filamentation gene expression response in filamentous mutants. The wild-type strain and the filamentous mutants were grown in SDG with or without 10% human serum for 6 h at 37°C prior to the isolation of total RNA, which was used for the determination of the relative levels of expression of the indicated genes. Asterisks indicate significant changes (*P* ≤ 0.05, two-tailed, unpaired Student’s *t* test) from the results seen with the wild-type strain grown in SDG with serum. Download FIG S2, TIF file, 1.9 MB. Copyright © 2020 Ruben et al. 2020 Ruben et al. This content is distributed under the terms of the Creative Commons Attribution 4.0 International license.

### Overexpression of *TUP1* does not inhibit hyphal morphology and expression of *ALS3* and *ECE1*.

**FIG 2:**
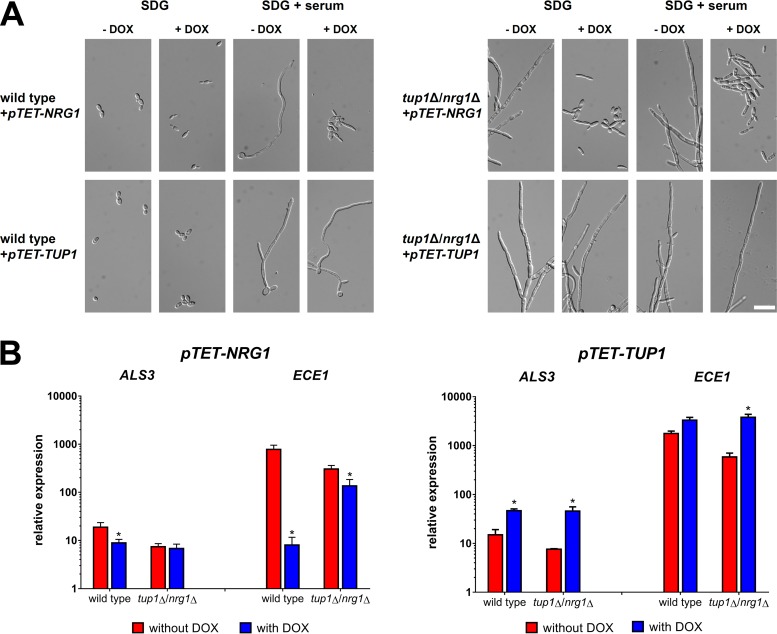
Overexpression of *TUP1* promotes *ALS3* and *ECE1* transcription. *TET* promoter-driven constructs of *NRG1* and *TUP1* were integrated into the *ADH1* loci of wild-type strain SC5314 and the *tup1*Δ/*nrg1*Δ double mutant. (A) The morphology of the resulting strains was studied by microscopy after 6 h of incubation at 37°C in SDG or SDG with 10% human serum with or without the addition of 50 μg/ml doxycycline. Scale bar, 20 μm. (B) Total RNA of strains expressing either the *pTET-TUP1* or the *pTET-NRG1* construct was isolated after 6 h growth in SDG with 10% human serum. The *pTET* constructs were activated by the addition of 50 μg/ml doxycycline to the medium. The isolated RNA was used for RT-qPCR to determine the relative levels of gene expression of *ALS3* and *ECE1*. A control RNA (wild type, 5 h YPD, 37°C) and the housekeeping gene *ACT1* were used for normalization. Asterisks mark significant differences after overexpression of either *NRG1* or *TUP1* compared to the same strain without overexpression (*P* ≤ 0.05, two-tailed, unpaired Student's *t* test).

Due to the surprising finding that Tup1 was required for high-level *ALS3* and *ECE1* expression, we studied the effects of induced overexpression of *TUP1* and *NRG1* using *pTET* constructs. Overexpression of *NRG1*, but not *TUP1*, prevented filamentation in a wild-type background (Fig. 2A). Both *pTET* constructs were also integrated into the *tup1*Δ/*nrg1*Δ double mutant. Again, overexpression of *NRG1* triggered filament-to-yeast reversion whereas overexpression of *TUP1* did not affect filamentous growth (Fig. 2A). Induced overexpression of *NRG1* in the wild type led to decreased *ALS3* and *ECE1* transcript levels (Fig. 2B). In contrast, *TUP1* overexpression enhanced the expression of both genes and even restored high mRNA levels of *ECE1* in the *tup1*Δ/*nrg1*Δ mutant (Fig. 2B). Nonetheless, the presence of Tup1 was important for the repression of *ECE1* by *NRG1* overexpression (Fig. 2B), illustrating that it contributes to repression and activation of this gene.

### Ahr1 is required for the expression of *ECE1* and *ALS3*.

**FIG 3:**
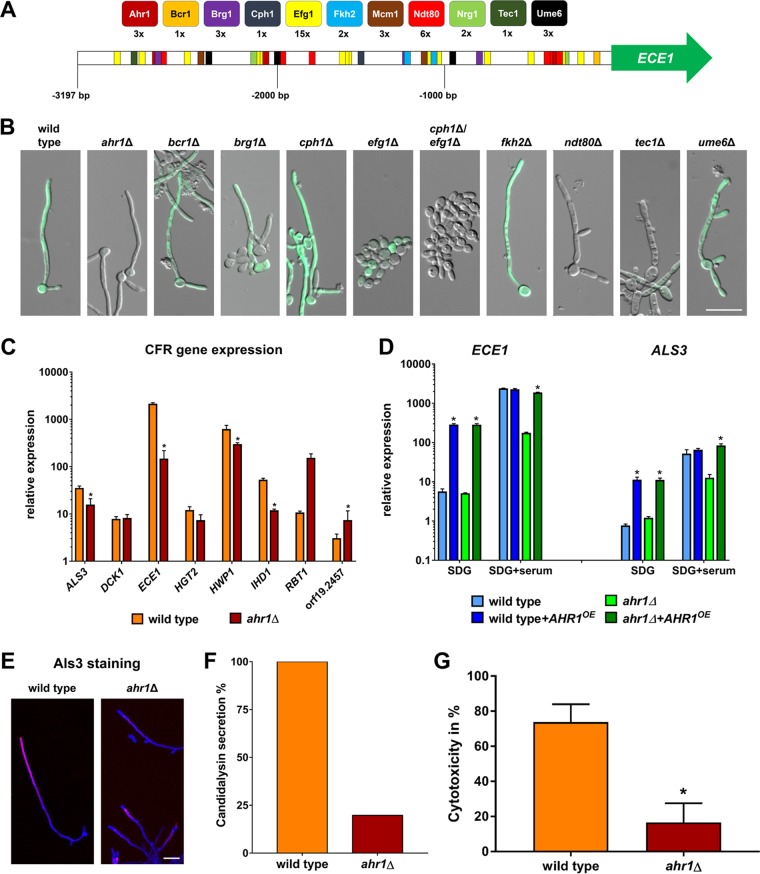
Ahr1 is required for high-level expression of *ECE1* and *ALS3*. (A) Scheme of the intergenic region upstream of *ECE1* with potential binding sites of transcriptional activators. (B) The wild-type strain and regulatory mutants expressing the *pECE1-GFP* construct were grown for 6 h at 37°C in SDG with 10% human serum prior to microscopy. Shown are the overlays of the DIC and the GFP channels. Scale bar, 20 μm. (C) The wild type and the *ahr1*Δ mutant were grown for 6 h at 37°C in SDG medium with serum prior to isolation of total RNA, which was used for the determination of relative gene expression levels. Asterisks indicate significant changes (*P* ≤ 0.05, two-tailed, unpaired Student's *t* test) compared to the wild type. (D) Total RNA of the wild-type and *ahr1*Δ strains with or without *AHR1* overexpression. The strains were grown for 6 h at 37°C in SDG with or without 10% human serum for the determination of relative gene expression levels. Asterisks indicate significant changes (*P* ≤ 0.05, two-tailed, unpaired Student's *t* test) compared to the strain without *AHR1* overexpression. (E) Cells of the indicated strains were grown for 6 h in SDG with 10% human serum at 37°C and were then stained with a monoclonal anti-Als3 antibody (pink signal), followed by a second staining with calcofluor white (blue signal). Shown are the overlays of the images taken in the Cy5 and DAPI channels. Scale bar, 20 μm. (F) Levels of candidalysin secretion of the wild type and the *ahr1*Δ mutant were measured by mass spectrometry after 18 h growth in YNBS (pH 7.2). Candidalysin contents measured for wild-type hyphae were defined as 100%. (G) Cytotoxicity of the indicated strains was determined by LDH release from the infected TR-146 cells after a 24 h coincubation. Asterisks indicate significant changes (*P* ≤ 0.05, two-tailed, unpaired Student's *t* test) compared to the wild type.

In addition to the influence of putative repressors, we also examined the influence of putative activators on the expression of *ECE1*. The 5′ intergenic region of the gene has a size of 3,197 bp and contains potential binding motifs for a variety of transcriptional activators which could at least in theory bind and regulate gene expression (Fig. 3A; see also Table S1). To visualize *ECE1* expression, GFP was integrated into the *ECE1* loci of mutants lacking these transcription factors to monitor *ECE1* expression levels. The resulting strains were then examined for GFP signals under hyphal growth conditions. The *efg1*Δ, *cph1*Δ/*efg1*Δ, *ndt80*Δ, and *tec1*Δ mutants were not able to form hyphae, and in accordance with this phenotype, the GFP signal was mostly absent or weak (Fig. 3B). Strains lacking *BCR1* or *BRG1* partially formed hyphae with GFP signals present (Fig. 3B). The hyphae of the *cph1*Δ and *fkh2*Δ mutants showed bright GFP signals (Fig. 3B). The *ume6*Δ mutant initially formed hyphae with the expected GFP signal, although the hyphal growth quickly reverted to yeast growth (Fig. 3B). The only mutant which was able to form morphologically normal hyphae without a GFP signal was the *ahr1*Δ mutant (Fig. 3B). RT-qPCR results confirmed the microscopic observations and showed that the level of *ECE1* transcription in *ahr1*Δ filaments was indeed significantly lower than in wild-type hyphae (Fig. 3C; see also Fig. S3). As seen with *ECE1*, the levels of transcription of *ALS3*, *HWP1*, and *IHD1* were significantly reduced in *ahr1*Δ hyphae (Fig. 3C). Reintegration of *AHR1* into the *ADH1* locus, which also led to overexpression of the *AHR1* gene, restored the high-level expression of *ALS3* and *ECE1* in *ahr1*Δ hyphae (Fig. 3D). Interestingly, p*ADH1*-driven *AHR1* induced upregulation of *ALS3* and *ECE1* in the wild type already under yeast growth conditions (Fig. 3D). As observed for the *tup1*Δ mutant, the intermediate expression levels of the *ALS3* and *ECE1* genes in the *ahr1*Δ mutant correlated with an absence of Als3 protein surface localization (Fig. 3E) and low secretion of candidalysin (Fig. 3F). In agreement with these findings, the level of virulence of the mutant in an oral epithelial cell infection model was found to be attenuated (Fig. 3G).

10.1128/mBio.00206-20.3 FIG S3 *ECE1* transcription in activator mutants. The wild-type strain and the indicated activator mutants were grown in SDG medium with 10% human serum for 4 h prior to the isolation of total RNA. The RNA was used to determine the relative levels of expression of *ECE1*. Asterisks indicate significant changes (*P* ≤ 0.05, two-tailed, unpaired Student’s *t* test) compared to the wild type. Download FIG S3, TIF file, 0.9 MB. Copyright © 2020 Ruben et al. 2020 Ruben et al. This content is distributed under the terms of the Creative Commons Attribution 4.0 International license.

10.1128/mBio.00206-20.8 TABLE S1 Putative transcription factor binding sites in the 5′ intergenic region of *ECE1*. Download Table S1, XLSX file, 0.01 MB. Copyright © 2020 Ruben et al. 2020 Ruben et al. This content is distributed under the terms of the Creative Commons Attribution 4.0 International license.

### *MCM1* overexpression can induce the expression of *ECE1* and *ALS3* in the presence of Ahr1 and Tup1.

**FIG 4:**
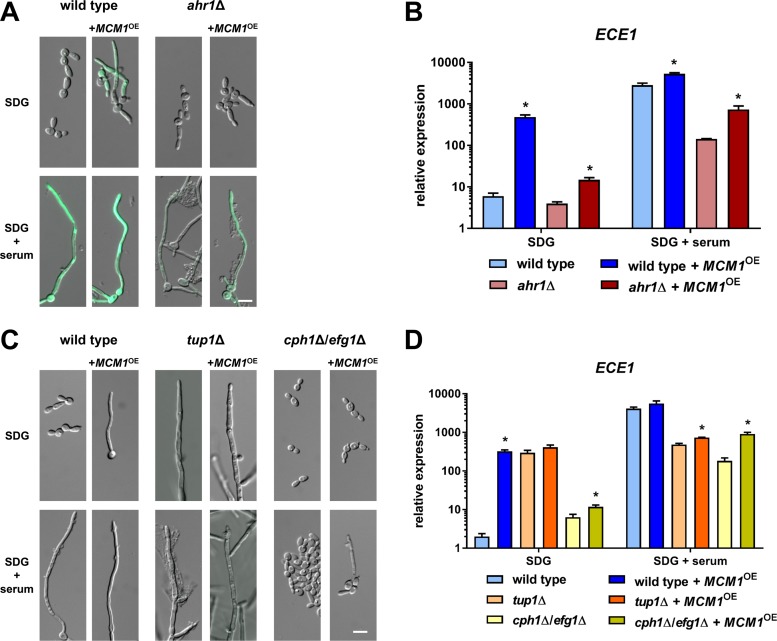
*MCM1* overexpression activates transcription of *ECE1*. (A) The wild type and the *ahr1*Δ mutant with integrated *pECE1-GFP* and with or without *pADH1-MCM1* were grown in SDG medium with or without 10% human serum for 6 h at 37°C prior to microscopy. Scale bar, 20 μm. (B and D) Total RNA from the wild-type and *ahr1*Δ mutant strains (B) and from the wild-type and *tup1*Δ and *cph1*Δ/*efg1*Δ mutant strains was isolated after 6 h growth and used to determine the relative *ECE1* expression levels. Scale bars, 20 μm. Asterisks indicate significant changes (*P* ≤ 0.05, two-tailed, unpaired Student's *t* test) compared to the corresponding strain without *MCM1* overexpression. (C) The indicated strains were grown for 6 h at 37°C in SDG medium with or without 10% human serum prior to microscopy. Scale bar, 10 μm.

Ahr1 is known for its interaction with Mcm1, a transcription factor which itself also binds to the *ECE1* promoter (28, 29). As deletion of this essential transcriptional regulator is not feasible, we analyzed the effects of *MCM1* overexpression under the control of the *ADH1* promoter in the wild-type and *ahr1*Δ strains, each carrying the *ECE1*-GFP reporter system. This overexpression resulted in observations of GFP signals under yeast and hyphal growth conditions in the wild-type background (Fig. 4A). In the *ahr1*Δ mutant, however, the *MCM1* overexpression led to a GFP signal in hyphae only (Fig. 4A). RT-qPCR showed that *MCM1* overexpression triggered a strong upregulation of *ECE1* in the wild-type background under both growth conditions (Fig. 4B). This effect of the overexpression was much lower in the absence of Ahr1 under yeast growth conditions (Fig. 4B). However, under hyphal growth conditions, *MCM1* overexpression significantly increased *ECE1* transcription in the *ahr1*Δ mutant, although not up to wild-type hyphal levels (Fig. 4B). Due to these observations, we studied the effects of *MCM1* overexpression in the filamentous *tup1*Δ mutant and the nonfilamentous *cph1*Δ/*efg1*Δ double mutant. *MCM1* overexpression partially rescued filamentation in the *cph1*Δ/*efg1*Δ double mutant (Fig. 4C), which was associated with upregulation of *ECE1*, although not to the level found in wild-type hyphae (Fig. 4D). In contrast, it was unable to compensate for the absence of Tup1 either morphologically or regarding *ECE1* expression, which remained at intermediate levels (Fig. 4D). Similar gene expression patterns were observed for *ALS3*, *HWP1*, and *IHD1* (Fig. S4).

10.1128/mBio.00206-20.4 FIG S4 Core filamentation gene expression response during overexpression of *MCM1*. The indicated strains were grown for 4 h at 37°C in SDG medium with or without 10% human serum prior to the isolation of total RNA. The RNA was used to determine the relative levels of gene expression. Asterisks indicate significant changes (*P* ≤ 0.05, two-tailed, unpaired Student’s *t* test) compared to the wild type. Download FIG S4, TIF file, 2.6 MB. Copyright © 2020 Ruben et al. 2020 Ruben et al. This content is distributed under the terms of the Creative Commons Attribution 4.0 International license.

### Hyperactive Ahr1 induces *ALS3* and *ECE1* expression without environmental stimuli by direct binding to the target promoters.

**FIG 5:**
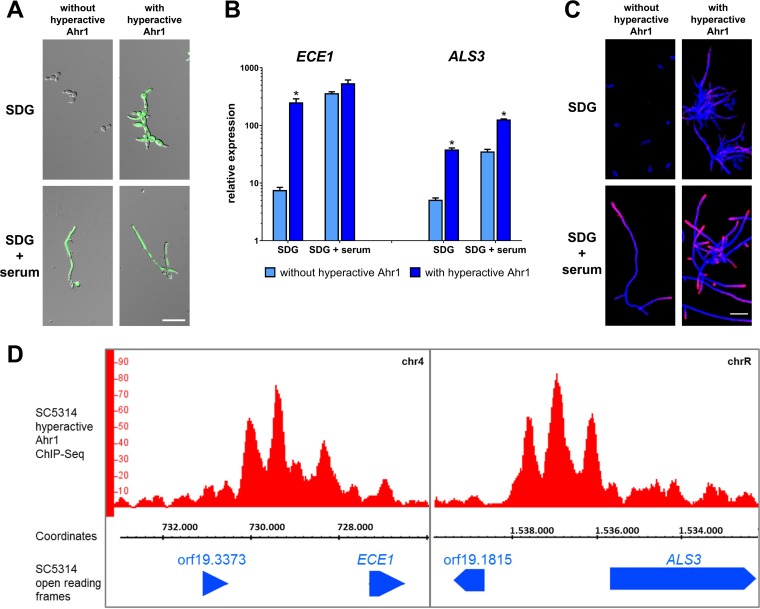
Hyperactive Ahr1 induces high-level expression of *ALS3* and *ECE1*. (A) The indicated strains with or without the hyperactive Ahr1 were grown in SDG or SDG with 10% human serum. Pictures were taken after 6 h of growth at 37°C. Shown are the overlays of the DIC and the GFP channels. Scale bar, 20 μm. (B) After 6 h of growth, total RNA of the strains grown as described for panel A was isolated and used for determination of relative gene expression levels. Asterisks indicate significant changes (*P* ≤ 0.05, two-tailed, unpaired Student's *t* test) in mutants with hyperactive Ahr1 compared to their background strains without the hyperactive allele. (C) Cells of the indicated strains were grown for 6 h in SDG with 10% human serum at 37°C and were then stained with a monoclonal anti-Als3 antibody (pink signal), followed by a second staining with calcofluor white (blue signal). Shown are the overlays of the images taken in the Cy5 and DAPI channels. Scale bar, 20 μm. (D) ChIP-Seq shows direct binding of hyperactive Ahr1 to the promoters of *ECE1* and *ALS3*. Genomic DNA used for ChIP-Seq was isolated from the wild-type strain with hyperactive Ahr1 after 6 h growth in SDG medium at 37°C. The binding peaks as shown in the IGB viewer are displayed.

It was previously shown that a hyperactive version of Ahr1 can trigger filamentation in C. albicans (30). This construct, consisting of an *AHR1* allele which was fused to a Gal4 activator domain (GAD) and a 3× hemagglutinin (HA_3_) tag, was integrated into the *ADH1* locus of a wild-type derivative already carrying the *pECE1-GFP* construct. The resulting strain showed a bright GFP signal already under yeast growth conditions (Fig. 5A). Independently of the environment, the expression of *ECE1* and *ALS3* in the wild type with hyperactive Ahr1 was as high as in normal wild-type hyphae (Fig. 5B). Thus, addition of the Gal4 activator domain further enhanced the effects of *AHR1* overexpression (Fig. 3D). We also observed slightly increased Als3 protein signals in the wild type with hyperactive Ahr1 under yeast growth conditions (Fig. 5C).

To test whether induction of *ECE1* was related to promoter binding of Ahr1, we used the wild-type derivative with the hyperactive Ahr1 to perform chromatin immunoprecipitation sequencing (ChIP-Seq) experiments under yeast growth conditions. ChIP-Seq provided clear evidence of physical binding of the hyperactive Ahr1 to the promoters of *ECE1* and *ALS3* (Fig. 5D) but also to those of other CFR genes (Table S2). The Ahr1 binding motif was found to be identical to one previously described (31). A nontagged version of the hyperactive Ahr1 was used to validate the ChIP-Seq results as shown for the *ECE1* neighborhood (Fig. S5).

10.1128/mBio.00206-20.5 FIG S5 Validation of ChIP-Seq results for wild-type derivatives with hyperactive Ahr1. ChIP-Seq reads (red) mapped to the genome of the SC5314 wild type (ASM18296v3). An extract from chromosome 4 with the ORFs of *ECE1*, *RBT1*, and HWP1 (blue) is shown. (Top) ChIP-Seq reads of the control strain with hyperactive Ahr1 without a HA_3_ tag in the SC5314 background. (Middle) ChIP-Seq reads of the mutant with hyperactive Ahr1 with a HA_3_ tag in the SC5314 background. Identified peak summits are depicted in black. Download FIG S5, TIF file, 0.9 MB. Copyright © 2020 Ruben et al. 2020 Ruben et al. This content is distributed under the terms of the Creative Commons Attribution 4.0 International license.

10.1128/mBio.00206-20.9 TABLE S2 Results of the ChIP-Seq analysis performed with hyperactive Ahr1. Download Table S2, XLSX file, 0.03 MB. Copyright © 2020 Ruben et al. 2020 Ruben et al. This content is distributed under the terms of the Creative Commons Attribution 4.0 International license.

### Hyperactive Ahr1 binds to genes encoding hypha-associated transcription factors and induces their expression.

**FIG 6:**
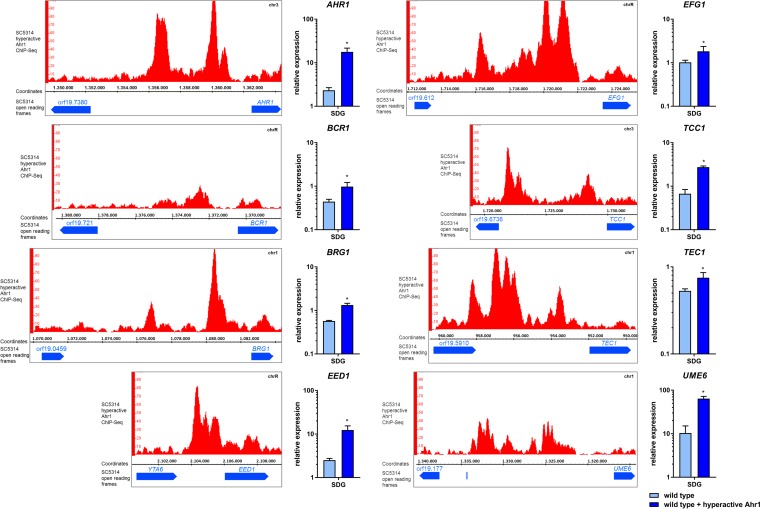
Binding of hyperactive Ahr1 increases expression levels of regulatory genes. The wild-type strain with hyperactive Ahr1 was grown for 4 h in SDG medium at 37°C prior to the ChIP-Seq analyses. The binding of hyperactive Ahr1 to regulatory genes is displayed in IGB viewer images. After growth under the same conditions, total RNA of the wild type with or without hyperactive Ahr1 was isolated at the same time point and used for the determination of relative gene expression levels. Asterisks indicate significant changes (*P* ≤ 0.05, two-tailed, unpaired Student's *t* test) in the presence of hyperactive Ahr1 compared to the wild type alone.

Hyperactive Ahr1 also bound to the promoters of transcriptional regulator genes *BCR1*, *BRG1*, *EFG1*, *TEC1*, and *UME6*, and this binding correlated with their upregulation (Fig. 6; see also Table S2). We also detected binding to the promoter of *EED1*, which was associated with increased expression of the gene (Fig. 6; see also Table S2). In addition, hyperactive Ahr1 bound to the promoter of *TCC1*, which encodes a Tup1-interacting protein (Fig. 6; see also Table S2). However, physical attachment to the promoters of *NRG1* and *TUP1* was not detected. Binding of hyperactive Ahr1 to the promoter of the *AHR1* gene itself might indicate a self-controlling feedback mechanism (Fig. 6; see also Table S2).

### Hyperactive Ahr1 induces *ALS3* and *ECE1* expression in the absence of Cph1 and Efg1 but depends on Tup1.

**FIG 7:**
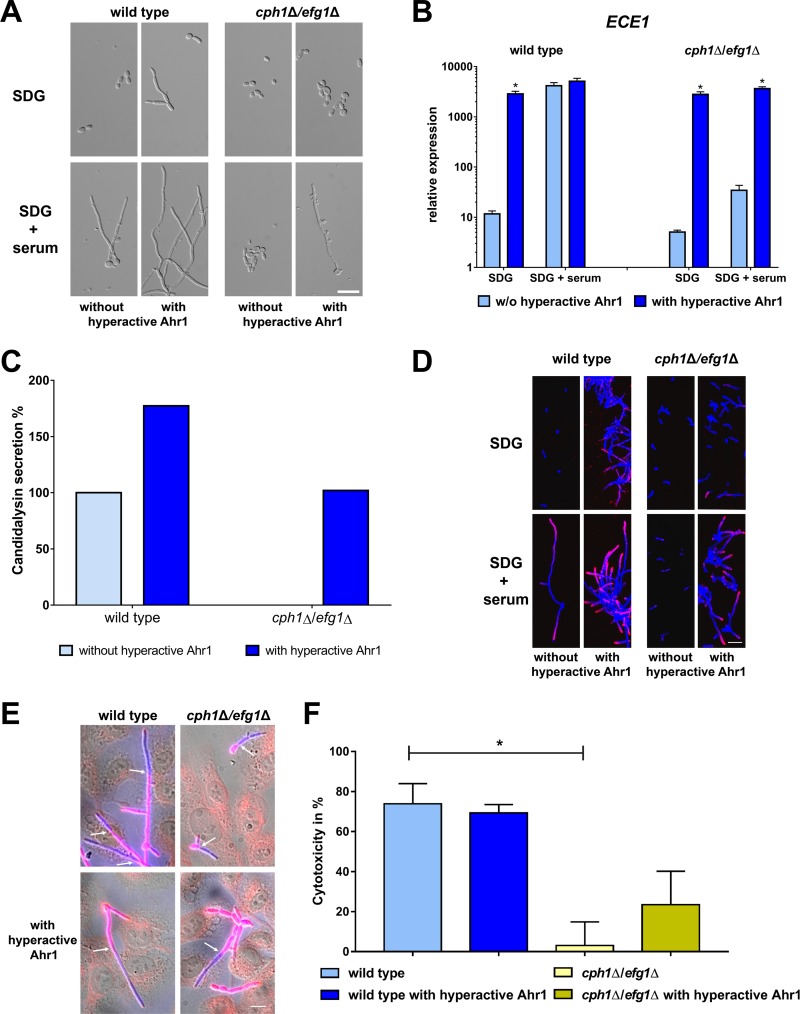
Hyperactive Ahr1 activates *ECE1* and *ALS3* expression in the nonfilamentous *cph1*Δ/*efg1*Δ mutant. (A) Wild type SC5314 and *cph1*Δ/*efg1*Δ mutant with or without the hyperactive Ahr1 were grown for 6 h at 37°C in either SDG or SDG with 10% human serum prior to microscopy. Scale bar, 20 μm. (B) After 6 h growth under the same conditions as described for panel A, total RNA of these strains was isolated and used for determination of relative *ECE1* expression levels. Asterisks indicate significant changes (*P* ≤ 0.05, two-tailed, unpaired Student's *t* test) in mutants with hyperactive Ahr1 compared to their background strains without the hyperactive allele. (C) Candidalysin secretion of wild-type and *cph1*Δ/*efg1*Δ strains with or without the hyperactive Ahr1 was measured by LC-MS/MS after 18 h growth in YNBS (pH 7.2). Candidalysin secreted by wild-type hyphae was defined as 100%. (D) Cells of the indicated strains were grown for 6 h in SDG with 10% human serum at 37°C and then stained with the Als3 antibody (pink) and calcofluor white (blue signal). Shown are the overlays of the images taken in the Cy5 and DAPI channels. Scale bar, 20 μm. (E) TR-146 oral epithelial cells infected with wild-type and *cph1*Δ/*efg1*Δ strains with or without hyperactive Ahr1 after 4 h coincubation. C. albicans outside human cells is shown in pink; C. albicans inside human cells is shown in blue. Arrows mark the point of invasion. Scale bar, 20 μm. (F) Cytotoxicity of the indicated strains was determined by the release of LDH from infected TR-146 cells after a 24 h coincubation. Asterisks mark significant changes (*P* ≤ 0.05, two-tailed, unpaired Student's *t* test) to values of the wild-type strain.

In the next step, we integrated the allele coding for the hyperactive Ahr1 into the nonfilamentous *cph1*Δ/*efg1*Δ double mutant. This partially restored filamentation in the double mutant (Fig. 7A) and, more strikingly, induced a significant increase of *ECE1* transcription to the levels in wild-type hyphae (Fig. 7B). In accordance with these observations, the *cph1*Δ/*efg1*Δ strain with hyperactive Ahr1 showed an increased secretion of candidalysin which was absent from the *cph1*Δ/*efg1*Δ supernatants (Fig. 7C). Hyperactive Ahr1 also triggered the upregulation of *ALS3*, *HWP1*, and *IHD1* in *cph1*Δ/*efg1*Δ under both yeast and hyphal growth conditions (Fig. S6). This upregulation was sufficient to ensure a surface localization of Als3 protein on the *cph1*Δ/*efg1*Δ mutant cells under hyphal growth conditions (Fig. 7D). Cells of the double mutant with or without hyperactive Ahr1 were also able to invade human oral epithelial cells, although not as efficiently as the wild type (Fig. 7E). Despite a modestly increased level of lactate dehydrogenase (LDH) release of infected epithelial cells, hyperactive Ahr1 could restore virulence capacity only partially in the *cph1*Δ/*efg1*Δ mutant as the level of cell damage was still much lower than for the wild type (Fig. 7F). This suggests that secretion of candidalysin alone is not sufficient for full virulence in this mutant.

10.1128/mBio.00206-20.6 FIG S6 Hyperactive Ahr1 induces expression of *ALS3*, *HWP1*, and *IHD1* in the *cph1*Δ/*efg1*Δ double mutant. Wild-type and *cph1*Δ/*efg1*Δ strains with or without integrated hyperactive Ahr1 were grown for 6 h at 37°C in SDG medium with or without 10% human serum prior to the isolation of total RNA. The RNA was used to determine the relative levels of expression of genes *ALS3*, *HWP1*, and *IHD1*. Asterisks indicate significant changes (*P* ≤ 0.05, two-tailed, unpaired Student’s *t* test) compared to the background strains without hyperactive Ahr1. Download FIG S6, TIF file, 0.9 MB. Copyright © 2020 Ruben et al. 2020 Ruben et al. This content is distributed under the terms of the Creative Commons Attribution 4.0 International license.

**FIG 8:**
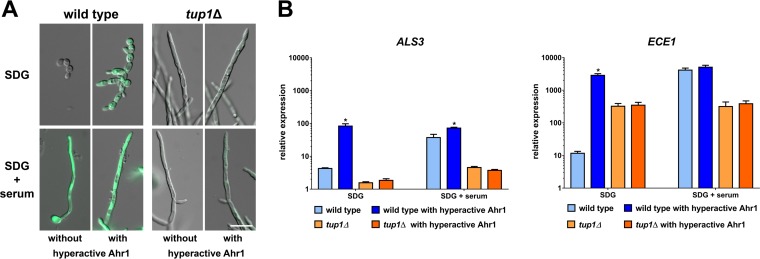
Beneficial effects of the hyperactive Ahr1 for *ALS3* and *ECE1* expression depend on Tup1. (A) The wild-type strain and *tup1*Δ derivates with integrated pECE1-GFP and with or without hyperactive Ahr1 were grown for 4 h in SDG medium with or without 10% human serum at 37°C prior to microscopy. Shown are overlays of the DIC and the GFP channels. Scale bar, 10 μm. (B) After the same time of incubation in the indicated media, total RNA of the indicated strains was isolated and used for RT-qPCR to determine the relative levels of gene expression of *ALS3* and *ECE1*. Asterisks indicate significant changes (*P* ≤ 0.05, two-tailed, unpaired Student's *t* test) in mutants with *AHR1-GAD* compared to their background strains without the hyperactive allele.

Interestingly, the hyperactive Ahr1 was able to induce a significant increase of *ECE1* transcription in mutants lacking other hyphae regulators, such as *ndt80*Δ, *tec1*Δ, and *ume6*Δ mutants (Fig. S7). However, the hyperactive Ahr1 did not enhance the expression of *ECE1* and *ALS3* in the *tup1*Δ mutant, indicating that the presence of Tup1 is essential for the upregulation of both genes by the hyperactive Ahr1 (Fig. 8).

10.1128/mBio.00206-20.7 FIG S7 Hyperactive Ahr1 restores *ECE1* transcription in regulatory mutants. (A) The indicated strains with or without integrated hyperactive Ahr1 were grown for 4 h at 37°C in SDG medium with or without 10% human serum prior to microscopy. Scale bar, 10 μm. (B) Total RNA from the aforementioned strains was isolated after the same time of growth. The RNA was used to determine the relative levels of expression of the *ECE1* gene. Asterisks indicate significant changes (*P* ≤ 0.05, two-tailed, unpaired Student’s *t* test) compared to the background strains without hyperactive Ahr1. Download FIG S7, TIF file, 2.6 MB. Copyright © 2020 Ruben et al. 2020 Ruben et al. This content is distributed under the terms of the Creative Commons Attribution 4.0 International license.

## DISCUSSION

The results of our study revealed a complex picture of how the virulence-associated genes *ALS3* and *ECE1* of C. albicans are transcriptionally regulated. Both genes have long been known for high mRNA abundance in hyphae, and we found that these high transcript levels in hyphae are indeed required for function, as even a drop to a more intermediate level of *ECE1* expression led to the absence of candidalysin secretion. A similar correlation was found between *ALS3* expression and localization of its encoded protein on the cell surface. This striking disparity between intermediate transcript levels and a lack of functional protein (surface Als3 or secreted candidalysin) may indicate posttranscriptional regulation of mRNA and/or protein levels that warrant further studies. Our study results show that Ahr1 and Tup1 are key contributors in this complex regulation of the two genes in the different C. albicans morphologies. The presence of both regulators is required to reach high transcription levels, revealing new functions for both of them. Here, we showed that Ahr1 binds directly to the promoters of *ALS3* and *ECE1* and induces their transcription. These findings expand the previous knowledge about Ahr1, which was thus far known for its involvement in white-opaque switching and in fungal modulation of the environmental pH but also in regulation of fungal adhesion and filamentation (28, 30–32). This novel role of Ahr1 might explain why the deletion mutant not only is attenuated in an oral epithelium infection model but also fails to induce pyroptosis in infected phagocytes (7, 33, 34), for which candidalysin plays an important role (8, 35). According to the Candida Genome Database, orthologs of Ece1 can be found in the genomes of C. dubliniensis and C. tropicalis, which are the closest relatives of C. albicans (36). Ahr1 homologs can similarly be found in both fungi, hinting at the possibility that the regulatory mechanism described here is conserved in other *Candida* species as well.

In previous works, native Ahr1 was C-terminally tagged and used for ChIP-based identifications of the binding motifs in the promoters of target genes (28, 31). Although the hyperactive Ahr1 which we used displayed enhanced activity compared to a native version, we identified the same binding motifs, indicating that the C-terminal addition of the Gal4 activator domain did not lead to false-positive results.

In addition, a hyperactive version of Ahr1 was found to be bound to the promoters of several transcription factor genes, including *BRG1*, *EFG1*, *TEC1*, and *UME6*, indicating that it regulates a variety of processes which are linked with hyphal growth and virulence. Interestingly, the hyperactive Ahr1 can restore *ALS3* and *ECE1* expression in deletion mutants of these transcription factors, indicating that it has a central role in the regulation of the two genes. In this context, we also observed that hyperactive Ahr1 restored high-level *ECE1* expression and candidalysin secretion in the nonfilamentous *cph1*Δ/*efg1*Δ mutant.

Hyperactive Ahr1 was not able to compensate for the absence of Tup1, in contrast to the aforementioned transcriptional activators. This illustrates the importance of the latter for high-level expression of *ALS3* and *ECE1*. Our results also indicate that Tup1 not only participates in the repression of both genes in yeast cells but also is required for their full activation in hyphae. This activating role represents a new concept for C. albicans Tup1 but is in agreement with previous findings from Saccharomyces cerevisiae. There, it was shown that Tup1 and its corepressor Cyc8/Ssn6 can contribute to both the repression and activation of several genes, including *GAL1* (37–41). In C. albicans, Tup1 might act as a backbone for the transcriptional control of *ALS3* and *ECE1* and change its interaction partners depending on whether repression or activation of the genes were required. We also observed that overexpression of *MCM1* induced the transcription of these two genes, an effect which was strongly dependent on Tup1 but less so on Ahr1. Further experiments will be required to determine whether Ahr1 can act alone or requires Mcm1 for its function and how Tup1 is involved in the recruitment of Ahr1 or of both transcription factors to their target promoters.

## MATERIALS AND METHODS

### C. albicans strains and media.

All C. albicans strains used in this study are listed in Table S3. Strains were routinely grown in YPD (20 g/liter glucose, 20 g/liter peptone, 10 g/liter yeast extract, with 20 g/liter agar if required) or SDG (synthetic defined glucose) minimal medium (20 g/liter glucose, 6.7 g/liter yeast nitrogen base [YNB] without amino acids; Sigma-Aldrich) at 37°C. For the induction of hyphal growth, strains were synchronized by two overnight incubations in SDG at 37°C. Cells (1 × 10^6^/ml) were then transferred to prewarmed SDG with 10% human serum (Sigma-Aldrich) and incubated at 37°C for the indicated times. If necessary, 50 μg/ml doxycycline (DOX) was added to the medium. For the medium shift, cells grown overnight in YPD at 30°C were diluted to 1 × 10^6^ cells/ml in prewarmed RPMI 1640 medium (Biochrom) or YPD and grown at 30°C (YPD) or 37°C (RPMI 1640). For the pH shift, overnight cultures of C. albicans strains grown in M199 medium (Sigma-Aldrich) (pH 4) were transferred to either M199 (pH 4) or M199 (pH 8) to reach a concentration of 1 × 10^6^ cells/ml and grown at 37°C.

10.1128/mBio.00206-20.10 TABLE S3 Strains, plasmids, and primers used in this study. Download Table S3, DOCX file, 0.02 MB. Copyright © 2020 Ruben et al. 2020 Ruben et al. This content is distributed under the terms of the Creative Commons Attribution 4.0 International license.

### Construction of plasmids.

All plasmids used in this study are listed in Table S3. The C. albicans *SAT1* (*caSAT1*) marker of the previously described pSK-pECE1-GFP-SAT1 plasmid (15) was excised with NotI and replaced by C. albicans *ARG4* from pFA-ARG4 (42) to generate plasmid pSK-pECE1-GFP-ARG4. For the construction of the pTET-TUP1 and pTET-NRG1 plasmids, *TUP1* and *NRG1* open reading frames (ORFs) were amplified from genomic C. albicans DNA with primers containing XhoI and BamHI restriction sites. The digested PCR product was then cloned into SalI-BglII-digested pNIM1 (43). To obtain an *NRG1* deletion cassette, *NRG1* upstream and downstream sequences were amplified with primers pairs NRG1P4/NRG1P5 and NRG1K/NRG1L, respectively, digested at the introduced restriction sites, and substituted for the *OPT5* sequences flanking the *SAT1* flipper cassette in the previously described pOPT5M2 plasmid (44) to generate pNRG1M2.

The previously described pECE1-GFP-SAT1 plasmid (45) was used for the creation of the *MCM1* overexpression construct. First, a 5′ region for the integration into the *ADH1* locus was amplified with primers 5′ADH1prom-KpnI-AscI-NarI and 3′-ADH1prom-XhoI and then cloned into NarI/XhoI-digested pECE1-GFP-SAT1, replacing the 5′ECE1 integration site. Second, a 3′ *ADH1* homology region which was amplified with primers 5′ADH1term-SacII and 3′ADH1term-SacI was cloned via SacII/SacI into this plasmid which then contained only *ADH1* homology regions *GFP* and *caSAT1*. In a final step, *MCM1* was amplified with the primers 5′MCM1-XhoI and 3′MCM1-EcoRV and cloned via XhoI/EcoRV into the constructed plasmid, replacing the GFP to create plasmid pADH1-MCM1-SAT1. For the creation of the *AHR1* overexpression construct, the *AHR1* gene was amplified from genomic C. albicans DNA using the primers 5′CaAHR1-XhoI and 3′AHR1-PmlI. After restriction performed with XhoI/PmlI, the *AHR1* ORF was cloned into the XhoI/EcoRV-restricted pADH1-GFP plasmid (45) plasmid, replacing the GFP and creating pADH1-AHR1-SAT1.

We used the already published pAHR1-GAD plasmid (30) to create a version without a HA_3_ tag. With the primers 5′XhoI-*AHR1* and 3′AflII-GAD, the *AHR1* ORF fused to the Gal4 activator domain was amplified from the pAHR1-GAD plasmid and restriction sites were introduced. Next, the DNA fragment was digested and ligated into the linearized vector (XhoI/AflII-digested pAHR1-GAD backbone).

Plasmids were validated by sequencing. Primers used for plasmid construction are listed in Table S3.

### Construction of C. albicans strains.

To generate a *tup1*Δ/*nrg1*Δ double mutant, both *NRG1* alleles were sequentially deleted in *tup1*Δ mutant BCa2-10 using the *SAT1*-flipping method (46) and the recyclable deletion cassette of plasmid pNRG1M2. Transformation cassettes were excised from the plasmids and cleaned up by gel extraction (QIAquick gel extraction kit; Qiagen). C. albicans strains were transformed by the use of the lithium acetate protocol (47) or by electroporation (48). Using the *caSAT1* gene as a selection marker, cells were incubated for 4 h in YPD at 30°C after the heat shock or electroporation and then plated on YPD with 200 μg/ml nourseothricin. Otherwise, transformants were grown on SDG agar plates. Transformants were validated by Southern hybridization (for the *tup1*Δ/*nrg1*Δ double mutant) or colony PCR (all other strains). The primers used for strain verification are listed in Table S3.

### Gene expression analysis.

Isolation of total fungal RNA was performed with a modified hot phenol chloroform method using zirconia beads to break up fungal cells. In short, cells were collected by centrifugation and resuspended in 500 μl AE-SDS buffer (including 10% SDS). A 500-μl volume of acid phenol chloroform-isoamyl alcohol and an amount of zirconia beads corresponding to a 500-μl volume were added. This mixture was then subjected to vortex mixing for 5 min and centrifuged. The supernatant was transferred to a new reaction tube, and further steps of isolation were performed as described previously (16). RT-qPCR and normalization against housekeeping gene *ACT1* and a control RNA (from SC5314 cells, grown for 5 h in YPD at 37°C) were performed as previously described (13). Relative gene expression levels were calculated according to the threshold cycle (Δ*C_T_*) method (49). The primers used for RT-qPCR are listed in Table S3 in the format R1-gene name for the forward primer and R2-gene name for the reverse primer.

### Fluorescence microscopy.

GFP fluorescence microscopy was performed with a Zeiss AxioObserver Z.1 microscope (Zeiss, Germany). The illumination time for the differential interference contrast (DIC) channel was 40 ms, and that for the GFP channel was 1,700 ms. The same illumination times were used for all samples to ensure that the GFP signals were comparable.

Immunofluorescence was performed with the anti-Als3 antibody and a Zeiss Z710 laser scanning microscope (Zeiss, Germany). Prior to microscopy, 1 × 10^6^ cells/ml of C. albicans strains were grown in SDG or SDG plus 10% human serum for 6 h in petri dishes with a glass bottom (μDish; Mobitec, Germany) at 37°C. After 6 h, cells were washed with 1× phosphate-buffered saline (PBS) and stained with the anti-Als3 antibody (1:500 diluted in 1× PBS) (11) for 60 min at room temperature. After an additional washing with 1× PBS, cells were incubated with a secondary DyLight649 goat anti-rabbit antibody (Thermo Scientific, Germany) or goat anti-rabbit-488 IgG antibody (Jackson Immuno Research, USA). Afterwards, cells were fixed with Histofix (Carl Roth, Germany) for 5 min and washed three times with 1× PBS. Finally, cells were stained with calcofluor white for 15 min. Z-stack image series of hyphae were taken using Zen software (Zeiss, Germany), and images were merged using the extended depth of focus settings.

### Ferritin growth assay.

The ferritin growth assay was performed as previously described (4).

### Candidalysin measurement.

Detection of secreted candidalysin was performed by liquid chromatography-tandem mass spectrometry (LC-MS/MS) with an Ultimate 3000 nano-RSLC system coupled to a QExactive Plus mass spectrometer (Thermo Fisher Scientific) as previously described (15). For hyphal growth conditions, strains were inoculated for 18 h in YNBS (75 mM MOPSO [3-morpholino-2-hydroxypropanesulfonic acid] buffer, 6.7 g/liter YNB, 20 g/liter sucrose, 1.1 g/liter *N*-acetyl-d-glucosamine, pH 7.2).

### Chromatin immunoprecipitation sequencing (ChIP-Seq).

Two C. albicans strains with a hyperactive Ahr1 (with and without C-terminal HA_3_-tag) in the SC5314 wild-type background were used for ChIP-Seq analyses, which were performed by Active Motif (USA). Formaldehyde fixation of the strains was performed after 6 h of incubation in SDG medium at 37°C following the company’s protocol. The strain without the HA_3_ tag was used for antibody validation to determine false positives. ChIP-Seq reads were analyzed by Active Motif, resulting in 325 peaks after peak calling and filtering the results. Peak data were further analyzed by BioControl Jena GmbH. Using the chromosomal position of the peaks, neighboring genes on both strands were identified. A sequence of 500 nucleotides centering at the maximum of each peak region was used as input for the online motif analysis tool Meme-ChIP (v. 5.1.0, 50), resulting in a highly significant, centrally enriched motif. Further confirmation was done with MochiView version 1.46 (51) and the Integrated Genome Browser (IGB) (52).

### Cytotoxicity and invasion assays.

Monolayers of TR146 buccal epithelial squamous cells, grown in Dulbecco’s modified Eagle’s medium/F12 (DMEM/F12) (Gibco) with 10% heat-inactivated fetal bovine serum, were infected with 4 × 10^4^ fungal cells and incubated in serum-free DMEM/F12 for 24 h at 37°C in 5% CO_2_ in 96-well plates. To determine cytotoxicity, lactate dehydrogenase (LDH) release in culture supernatants was measured using a cytotoxicity detection kit (Roche). Supernatant from Triton-X-treated, uninfected TR146 cells served as a full-lysis control, corresponding to 100% cytotoxicity. The experiment was repeated three times with biological triplicates in each round. The invasion assay was performed with the same cell line as previously described (27).
