# Supplementary figures and images for: Ahr1 and Tup1 Contribute to the Transcriptional Control of Virulence-Associated Genes in Candida albicans
Source: mBio. 2020 Apr 28;11(2):e00206-20. doi: 10.1128/mBio.00206-20 (PMC7188989; doi:10.1128/mBio.00206-20)

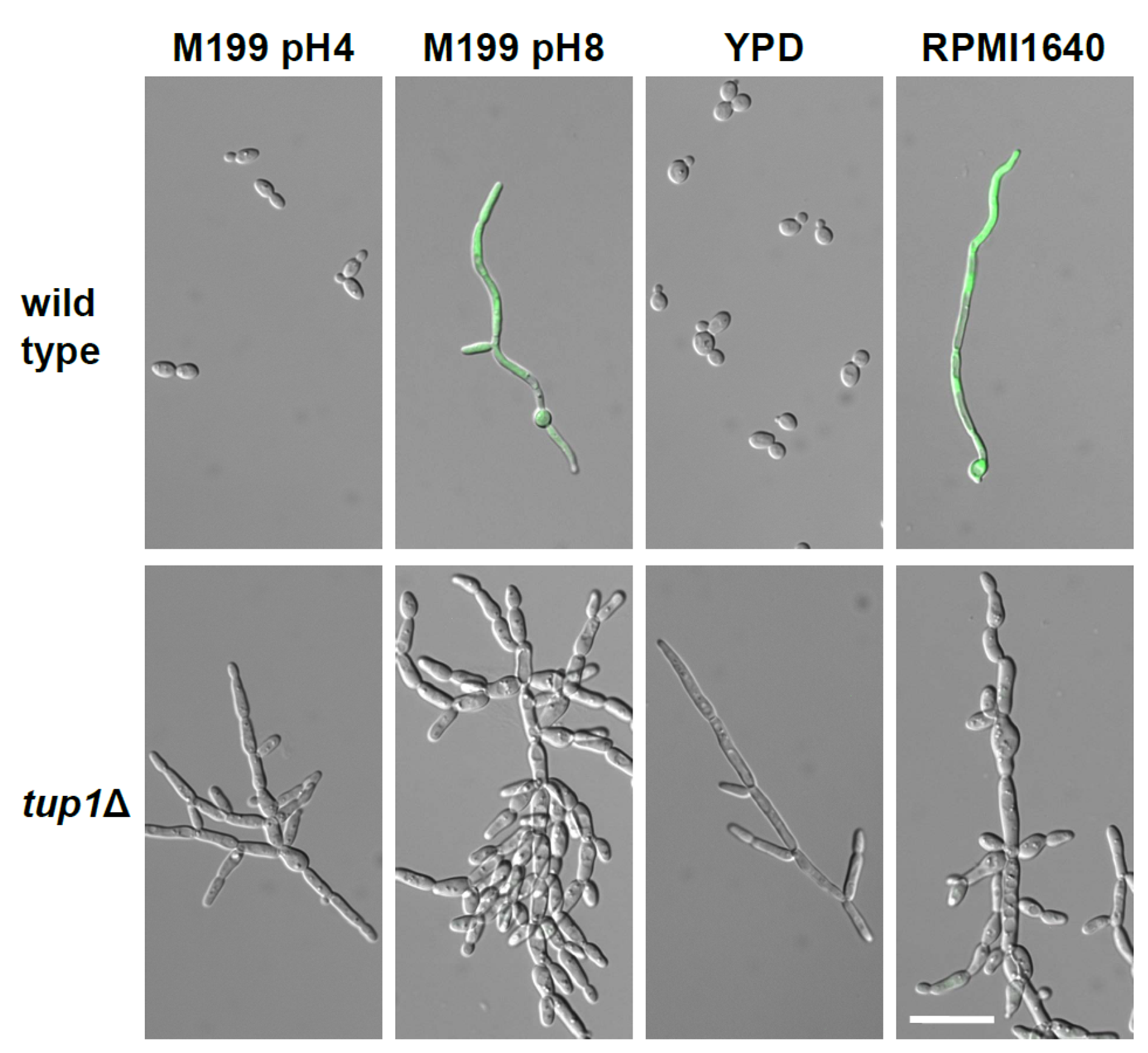

Supplement: FIG S1 [file mBio.00206-20-sf001.tif]

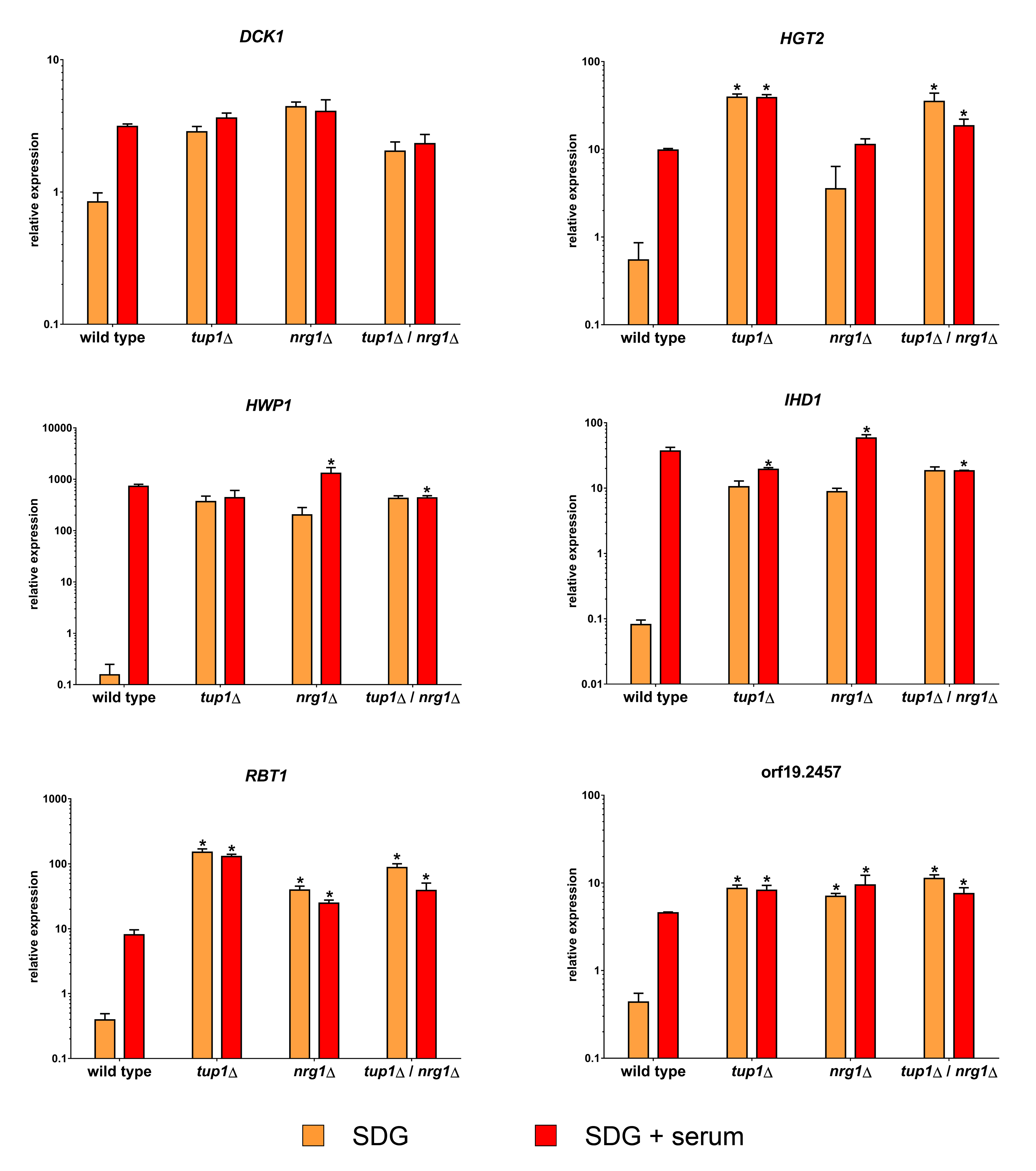

Supplement: FIG S2 [file mBio.00206-20-sf002.tif]

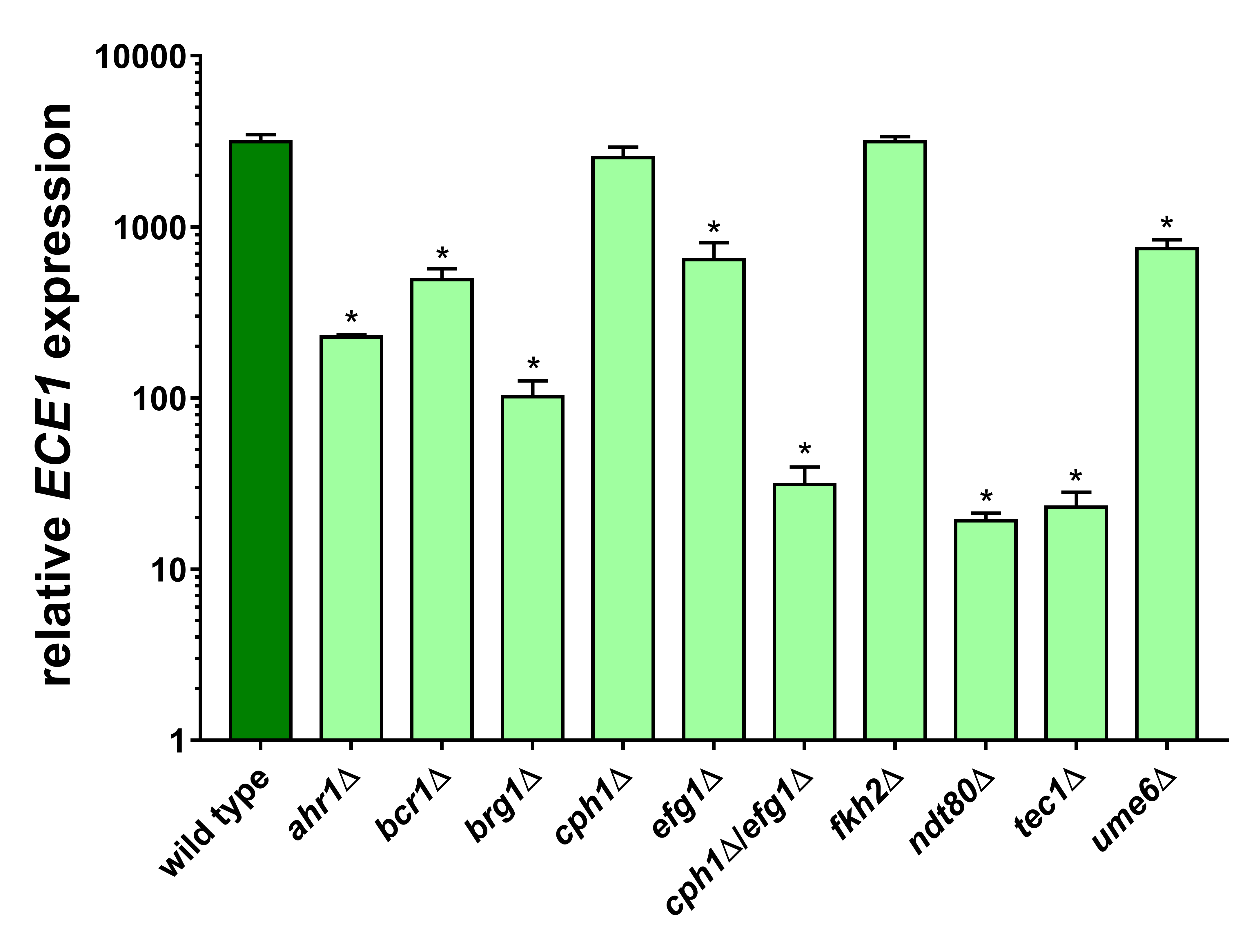

Supplement: FIG S3 [file mBio.00206-20-sf003.tif]

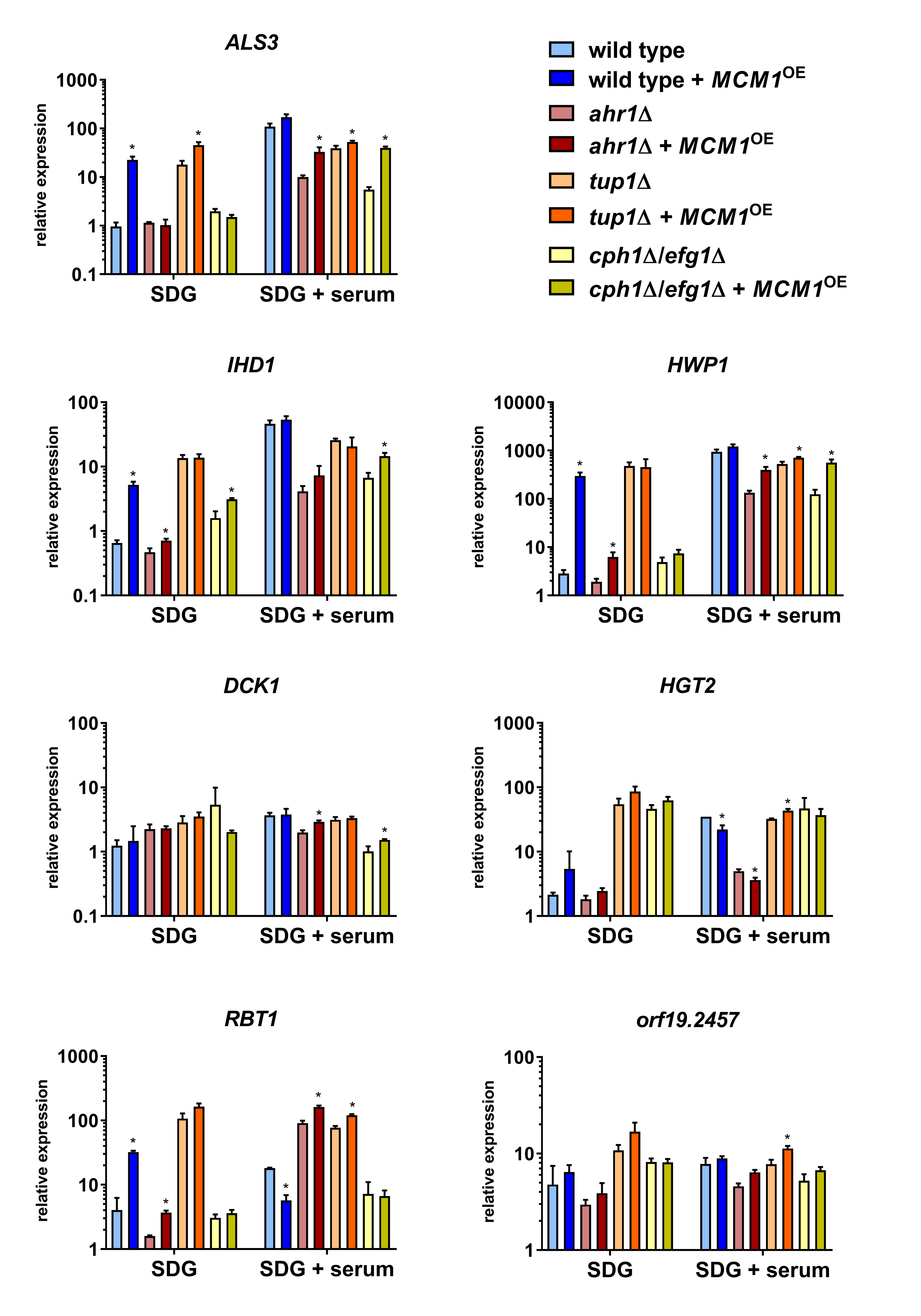

Supplement: FIG S4 [file mBio.00206-20-sf004.tif]

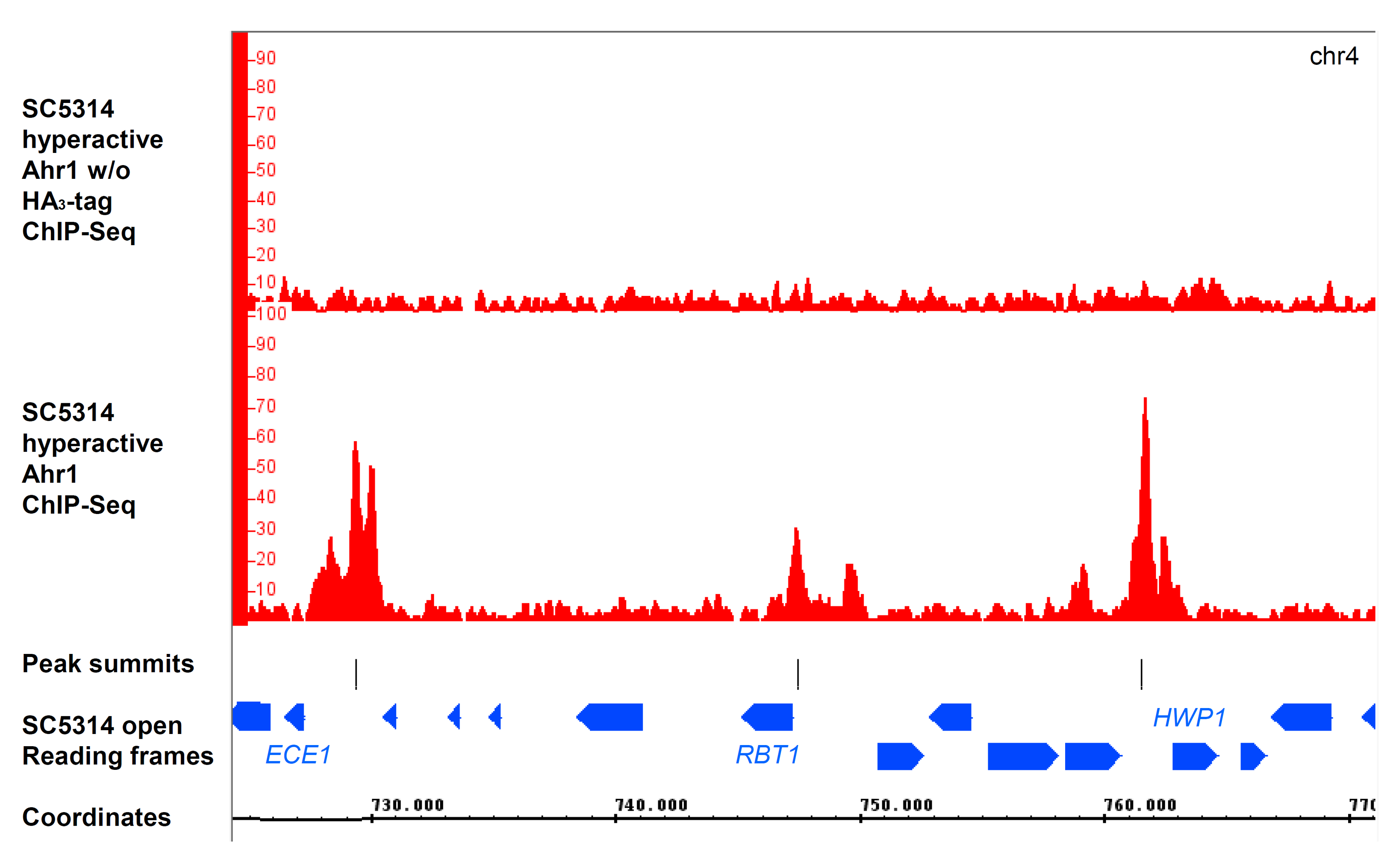

Supplement: FIG S5 [file mBio.00206-20-sf005.tif]

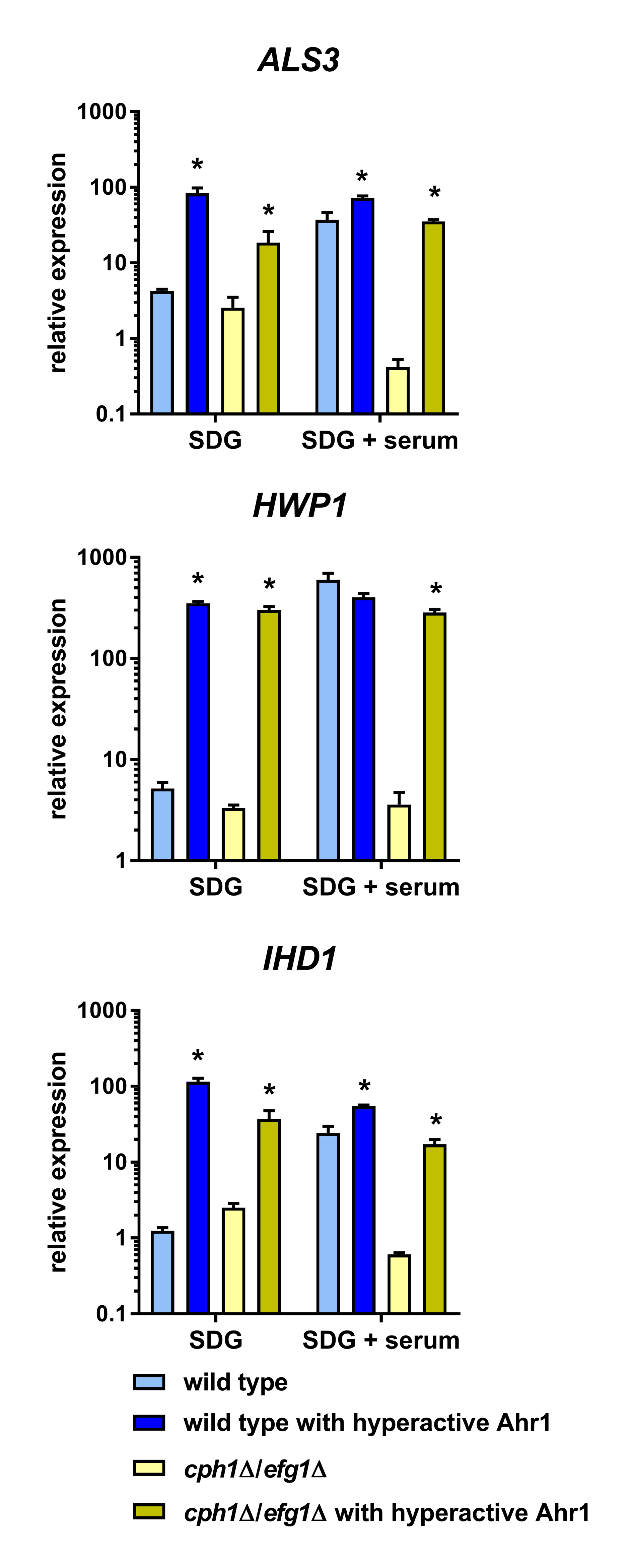

Supplement: FIG S6 [file mBio.00206-20-sf006.tif]
