# Supplementary material for: Ahr1 and Tup1 Contribute to the Transcriptional Control of Virulence-Associated Genes in Candida albicans
Source: mBio. 2020 Apr 28;11(2):e00206-20. doi: 10.1128/mBio.00206-20 (PMC7188989; doi:10.1128/mBio.00206-20)
Supplement: TABLE S3 [file mBio.00206-20-st003.docx]

**Dataset 3. Strains, plasmids and primers used in this study.**

**Table S3A. *C. albicans* strains used in this study.**

| **Strain** | **Genotype** | **Source** |
| --- | --- | --- |
| SC5314 | *C. albicans* wild type | 48 |
| SC5314 + *pECE1-GFP* | SC5314, *ECE1/ece1::GFP-caSAT1* | 12 |
| SN152 | *leu2Δ/leu2Δ +LEU2, his1Δ/his1Δ +HIS1,*  *arg4Δ/arg4Δ,URA3/ura3Δ::imm434, IRO1/iro1Δ::imm434* | Homann et al., 2009 |
| *ahr1*Δ | SN152, *ahr1::LEU2/ahr1::HIS1* | Homann et al., 2009 |
| *bcr1*Δ | SN152, *bcr1::LEU2/bcr1::HIS1* | Homann et al., 2009 |
| *brg1*Δ | SN152, *brg1::LEU2/brg1::HIS1* | Homann et al., 2009 |
| *cph1*Δ/*efg1*Δ | SC5314, *cph1::FRT/cph1::FRT, efg1::FRT/efg1::FRT* | Wartenberg et al., 2014 |
| *cph1*Δ | *CAI4, cph1::hisG/cph1::hisG-URA3-hisG* | Liu et al, 1994 |
| *efg1*Δ (HLC52) | *CAI4, efg1::hisG/ efg1::hisG-URA3-hisG* | Lo et al, 1997 |
| *fkh2*Δ | BWP17, *fkh2::FRT/fkh2::ARG4* | Greig et al, 2015 |
| *ndt80*Δ | SC5314, *ndt80::FRT/ndt80::FRT* | Sasse et al., 2011 |
| *nrg1*Δ+CIp10 | *CAI4, nrg1::hisG/ nrg1::hisG, RP10/rp10::CIp10 (URA3)* | Martin et al., 2011 |
| *nrg1*Δ | SN152, *nrg1::LEU2/nrg1::HIS1* | Homann et al., 2009 |
| *tec1*Δ | SN152, *tec1::LEU2/tec1::HIS1* | Homann et al., 2009 |
| *tup1*Δ+CIp10 | *CAI4, tup1::hisG/ tup1::hisG, RP10/rp10::CIp10 (URA3)* | Martin et al., 2011 |
| *tup1*Δ | SN152, *tup1::LEU2/tup1::HIS1* | Homann et al., 2009 |
| *ume6*Δ (UZ43) | *SN87*, *ume6::CdHIS1/ume6::CmLEU2* | Zeidler et al, 2008 |
| BCa2-10 | *ura3::imm434/ura3::imm434 tup1::hisG/tup1::hisG-URA3-hisG* | Braun and Johnson, 1997 |
| Δ*tup1*NRG1M (*tup1*Δ/*nrg1*Δ) | BCa2-10, *nrg1::FRT/nrg1::FRT* | This work. |
| *nrg1*Δ+CIp10+*pECE1-GFP* | *nrg1*Δ+CIp10, *ECE1/ece1::GFP-caSAT1* | This work. |
| *SC5314+pTET-NRG1* | SC5314, *ADH1/adh1::pTET-NRG1-caSAT1* | This work. |
| *tup1*Δ+CIp10+*pECE1-GFP* | *tup1*Δ+CIp10, *ECE1/ece1::GFP-caSAT1* | This work. |
| *tup1*Δ/*nrg1*Δ+*pECE1-GFP* | *tup1*Δ/*nrg1*Δ, *ECE1/ece1::GFP-caSAT1* | This work. |
| GC331 | SC5314, *ADH1/adh1::pTET-TUP1-caSAT1* | This work. |
| GC332 | *tup1*Δ/*nrg1*Δ, *ADH1/adh1::pTET-TUP1-caSAT1* | This work. |
| GC333 | *tup1*Δ/*nrg1*Δ, *ADH1/adh1::pTET-NRG1-caSAT1* | This work. |
| GC350 | SN152, *ECE1/ece1::GFP-ARG4* | This work. |
| GC351 | *ahr1*Δ, *ECE1/ece1::GFP-ARG4* | This work. |
| GC352 | *nrg1*Δ, *ECE1/ece1::GFP-ARG4* | This work. |
| GC355 | *tup1*Δ, *ECE1/ece1::GFP-ARG4* | This work. |
| GC364 | *bcr1*Δ, *ECE1/ece1::GFP-ARG4* | This work. |
| GC365 | *brg1*Δ, *ECE1/ece1::GFP-ARG4* | This work. |
|  | GC365, *ADH1/adh1::AHR1-GAD-HA_3_-caSAT1* | This work. |
| GC367 | *tec1*Δ, *ECE1/ece1::GFP-ARG4* | This work. |
|  | *tec1*Δ, *ADH1/adh1::AHR1-GAD-HA_3_-caSAT1* | This work. |
| GC371 | GC350, *ADH1/adh1::AHR1-GAD-HA_3_-caSAT1* | This work. |
| GC372 | GC355, *ADH1/adh1::AHR1-GAD-HA_3_-caSAT1* | This work. |
| GC374 | *cph1*Δ/ *efg1*Δ, *ADH1/adh1::AHR1-GAD-HA_3_-caSAT1* | This work. |
| GC416 | *cph1*Δ, *ECE1/ece1::GFP-caSAT1* | This work. |
| GC419 | *fkh2*Δ, *ECE1/ece1::GFP-caSAT1* | This work. |
|  | *efg1*Δ, *ECE1/ece1::GFP-caSAT1* | This work. |
|  | *cph1*Δ/ *efg1*Δ, *ECE1/ece1::GFP-caSAT1* | This work. |
| GC459 | *ndt80*Δ, *ECE1/ece1::GFP-caSAT1* | This work. |
| GC460 | *ndt80*Δ, *ADH1/adh1::AHR1-GAD-HA_3_-caSAT1* | This work. |
|  | *ume6*Δ, *ECE1/ece1::GFP-caSAT1* | This work. |
|  | *ume6*Δ, *ADH1/adh1::AHR1-GAD-HA_3_-caSAT1* | This work. |
| HFI12 | *ahr1Δ::FRT ahr1Δ::FRT* | This work. |
| HFI22 | HFI12, *ADH1/adh1::MCM1-SAT1* | This work. |
| HFI20 | SC5314, *ADH1/adh1::MCM1-SAT1* | This work. |
| GC393 | SC5314, *ADH1/adh1::AHR1-GAD-HA_3_-caSAT1* | This work. |
| GC467 | GC350, *ADH1/adh1::MCM1-SAT1* | This work. |
| *GC468* | GC351*, ADH1/adh1::MCM1-SAT1* | This work. |
| GC469 | SC5314, *ADH1/adh1::AHR1-GAD-w/o-HA_3_-caSAT1* | This work. |
| GC481 | *tup1*Δ, *ADH1/adh1::MCM1-SAT1* | This work. |
| GC489 | *cph1Δ/efg1Δ, ADH1/adh1::MCM1-SAT1* | This work. |
| HFI28 | SC5314, *ADH1*/*adh1*::*AHR1-SAT1* | This work |
| HFI30 | HFI12, *ADH1*/*adh1*::*AHR1*-*SAT1* | This work |

**Table S3B. Plasmids used in this study.**

| **Plasmid** | **Features** | **Method for tranformation** | **Source** |
| --- | --- | --- | --- |
| pSK-pECE1-GFP-SAT1 | *GFP*, *CaACT1* terminator, *CaSAT1* gene, homology regions for integration into *CaECE1* | *Asc*I/*Sac*I | Moyes et al., 2016 |
| pNIM1 | *Tet promoter,* homology regions for integration into *CaADH1, CaSAT1* gene , *GFP* ORF |  | Park and Morschhäuser, 2005 |
| pTET-NRG1 | *Tet promoter,* homology regions for integration into *CaADH1, CaSAT1* gene , *NRG1* ORF | *Kpn*I/*Sac*II | Park and Morschhäuser, 2005 |
| pAHR1-GAD | *AHR1* ORF without stop codon, fused with *GAL4* activator domain and 3xHa tag, *CaSAT1* gene, homology regions for integration into *CaADH1* | *Apa*I/*Sac*II | Schillig and Morschhäuser, 2013 |
| pFA-ARG4 | pFA-plasmid for deletion of *C. albicans* genes with *CaARG4* as seletion marker gene |  | Gola et al. 2003 |
| pOPT5M2 | Homology regions for deletion of *CaOPT5, SAT1* flipper cassette |  | Reuss and Morschhäuser 2006 |
| pNRG1M2 | Homology regions for deletion of *CaNRG1, SAT1* flipper cassette |  | This work |
| pSK-pECE1-GFP-ARG4 | *GFP*, *CaACT1* terminator, *CaARG4* gene, homology regions for integration into *CaECE1* | 5’ECE1prom-NarI/3’ECE1term-SacI | This work |
| pTET-TUP1 | *Tet promoter,* homology regions for integration into *CaADH1, CaSAT1* gene , *TUP1* ORF | *Kpn*I/*Sac*II | This work |
| pBluescript II SK + | Bluescript vector with multiple cloning site and ampicillin gene |  | Agilent |
| pAHR1-GAD w/o HA_3_ | *AHR1* ORF without stop codon, fused with *GAL4* activator domain, *CaSAT1* gene, homology regions for integration into *CaADH1* | *Apa*I/*Sac*II | Schillig and Morschhäuser, 2013 |
| pADH1-MCM1 | *MCM1*, *CaACT1* terminator, *CaSAT1* gene, homology regions for integration into *CaADH1* | *Asc*I/*Sac*I | This work |
| p*ADH1*-*AHR1* | *AHR1*, *CaACT1* terminator, *CaSAT1* gene, homology regions for integration into *CaADH1* | *Nar*I/ *Sac*I | This work |

**Table S3C. Oligonucleotide primers used in this study.**

| **Oligo-nucleotide** | **Sequence in 5‘ to 3‘ direction** |
| --- | --- |
| R1-ACT1 | TCAGACCAGCTGATTTAGGTTTG |
| R2-ACT1 | GTGAACAATGGATGGACCAG |
| R1-ALS3 | ATGGTCCTTATGAATCACCATCTA |
| R2-ALS3 | TAGCAGTTGTAGTTGTAGATGGAG |
| R1-ECE1 | ATCGAAAATGCCAAGAGAG |
| R2-ECE1 | AGCATTTTCAATACCGACAG |
| R1-BCR1 | ACTTTACCCCCAGTATCAAGCA |
| R2-BCR1 | ATCCAGTTTATTCACTACAACCATAG |
| R1-BRG1 | GGTTATTCCACGCTAAATTGGTAAAG |
| R2-BRG1 | ATGTGGCGATTCCTCCTTGTTG |
| R1-EED1 | TAGTGGTAATACCCAACGTG |
| R2-EED1 | CTGATATTTGAAATTTTGGAAGCTTTTC |
| R1-EFG1 | ACTAGTCCGGTAAATACCAAG |
| R2-EFG1 | TGTTGCTTTTGTCGTGCTGTG |
| R1-HGC1 | AGTCAGCTTCCTGCACC |
| R2-HGC1 | GATGAAGCAATACTAACTGCTGA |
| R1-NRG1 | GATCATGCCAAAATACGGTC |
| R2-NRG1 | TGTGAAGCTTCTAAAGTCCTG |
| R1-TEC1 | ACTTGCAACCACACCAAATGTG |
| R2-TEC1 | TTCGTGATATTTCCATATCCGGTATTC |
| R1-UME6 | TCTACTTCTAATCCAATGGTG |
| R2-UME6 | TATCATTACTTGATTTTTTCCGAG |
| pTET intern rev | ATATAATATAAATAGCACACACCCACAAC |
| 5'TUP1-XhoI* | TGAGctcgag*ATGTATCCCCAACGCACCCAGC* |
| 3'TUP1-BamHI* | GTCTggatcc*TTATTTTTTGGTCCATTTCCAAATTCTGGC* |
| 5'NRG1-XhoI* | TCAGctcgag*ATGCTTTATCAACAATCATATCC* |
| 3'NRG1-BamHI* | AGCTggatcc*CTATACTAGGCTCTTGGTGTTG* |
| pTET-CaAHR1-veri | TACTGGATTTGGCTCTAGATTGGGA |
| pTET intern rev | ATATAATATAAATAGCACACACCCACAAC |
| CaTUP1 intern veri rev | GTATTGTGACTTGTCGATAACCGA |
| pTET-NRG1 veri fwd | GATTGGTGCGACATTGGCTA |
| pTET-NRG1 veri rev | GTACTACCAGCAGCACTAGCA |
| CaAHR1 intern veri rev | GTTGTTGCTGCTGTTGCGGTTG |
| G1-ECE1 | CTCGCTGATTAGAGTTCAAGAGT |
| GFP veri rev | TGATCTGGGTATCTCGCAAAGCAT |
| R1-TUP1 | GACTTTGTTTTGTCGGTCTGCTG |
| G1-ADH1 | TATTCCGGAAGCTGGTAGCG |
| Mcm1-veri_rev | AGTGACCAAAGGTTGTAATTTAGG |
| I1-SAT1 | CGGTGATCCCTGAGCAGGTGGCG |
| G2-ADH1 | CCTAGTTGCCCTCCTTATGA |
| MCM1 intern | GACAGGTACTCAAGTGTTATTATTAGTTG |
| CaACT1term veri rev | GAATACAAAACCAGATTTCCAGATTTCCAG |
| 5'ECE1prom-NarI | GATCggcgccTCCAGCCACTATTTTGTACCTGT |
| 5'ECE1term-SacI | TCAGgagctcCGTTAAGAATATGAATGACAGTTGGTC |
| 3'ADH1prom-MluI-HIS-XhoI | GATACTCGAGatggtggtgatgatggtgCATacgcgt*AATTGTTTTTG* |
| KpnI-AscI-NarI-5‘ADH1prom | tcggggtaccggcgcgccggcgcc*GCCTTATCATTCTTCGTACAAG* |
| 3’MCM1-EcoRV | AGCTgatatc*TCATTGATATTGCTGTTGATTAGGTATG* |
| 5'MCM1-MluI | CATGacgcgt*ATGGCTATTAAAGAAGAAACAAATGAATTTAG* |
| NRG1K* | *CAACACCAAGAGcc*gcgg*ATAGATGTGGT* |
| NRG1L* | *GGCAATGCAAga*gctc*GCAAGATCATCGT* |
| 5'XhoI-AHR1 | CCATctcgag*ATGGCAAAGAAGAAACTAAATTCAACAATAAAG* |
| 3'AflII-GAD | ATGGcttaag*CTCTTTTTTTGGGTTTGGTGGGG* |
| 3'AHR1-PmlI* | ATGTcacgtg*TTAATCACTTACTGGGTGAATGTAGCGTACAG* |
| 5'CaAHR1-XhoI* | ACGActcgag*ATGGCAAAGAAGAAACTAAATTCAACAATA* |

* The homology region to the target gene is written in italics. Lower cases show introduced restriction sites that were used for further cloning steps.
